# Effect of Long Glass Fiber Orientations or a Short-Fiber-Reinforced Composite on the Fracture Resistance of Endodontically Treated Premolars

**DOI:** 10.3390/polym16091289

**Published:** 2024-05-04

**Authors:** Ecehan Hazar, Ahmet Hazar

**Affiliations:** 1 Department of Endodontics, Faculty of Dentistry, Zonguldak Bülent Ecevit University, Zonguldak 67600, Turkey; 2 Department of Restorative Dentistry, Faculty of Dentistry, Zonguldak Bülent Ecevit University, Zonguldak 67600, Turkey; dt.ahmethazar@yahoo.com.tr

**Keywords:** injectable composite, short-fiber-reinforced composite, unidirectional glass fiber, fracture resistance

## Abstract

This study aimed to evaluate the effect of direct restorations using unidirectional glass fiber orientations and a short-fiber-reinforced composite (SFRC) on the fracture resistance of endodontically treated premolars with mesio-occluso-distal cavities. Ninety double-rooted premolars were selected. Fifteen teeth were left intact/as a control group. The endodontic treatment and cavity preparations of seventy-five teeth were performed and divided into five experimental groups: Resin composite (RC), modified transfixed technique + RC, circumferential technique + RC, cavity floor technique + RC, and SFRC + RC. All teeth were fractured under oblique static loading at a 30° angle using a universal testing machine. The fracture patterns were observed and classified. Data were analyzed with one-way analysis of variance, Pearson chi-square, and Tukey HSD post hoc tests (*p* = 0.05). The highest fracture strength values were obtained in intact teeth (599.336 N), followed by modified transfixed + RC treated teeth (496.58 N), SFRC + RC treated teeth (469.62 N), RC (443.51 N), circumferential + RC treated teeth (442.835 N), and cavity floor + RC treated teeth (404.623 N) (*p* < 0.05). There was no significant difference between the RC and the circumferential technique + RC (*p* > 0.05). Unrepairable fractures were observed at low rates (20%) in the modified transfixed + RC and SFRC + RC teeth, and at higher rates in RC (73.3%), cavity floor + RC (60%), and circumferential + RC (80%) teeth. The application of an SFRC or the modified transfixed technique yielded an improved fracture strength and the fracture pattern of ETPs being restored with a universal injectable composite.

## 1. Introduction

The restoration of endodontically treated premolars (ETPs) with mesio-occluso-distal (MOD) cavities is a challenging treatment in conservative dentistry [1]. Irrigant solutions such as ethylenediaminetetraacetic acid and sodium hypochlorite used during endodontic treatment can reduce the mechanical resistance of teeth by altering the calcium to phosphate ratio [2]. The removal of the extensive tooth structure to prepare the endodontic access cavity, decreased collagen cross-linking, and the deficit of a protective feedback mechanism reduce the strength of dental tissues [3]. Therefore, cracks or vertical fractures occur more frequently in endodontically treated teeth than in vital teeth [4]. The fracture strength of premolars reduces by 46% with the loss of one marginal ridge and by 63% with the loss of both marginal ridges [5]. In addition, maxillary premolars are more prone to vertical fractures than molar teeth due to their exposure to high masticatory forces; therefore, it is recommended that maxillary ETPs with MOD cavities be restored without delay to preserve the remaining tooth structure and fulfill aesthetic and functional requirements [6,7].

Different materials and techniques including post and core, partial or full crowns, direct composite, amalgam, or ceramic restoration can be applied for the ETPs. The cuspal coverage technique has been stated as a successful indirect technique to prevent the cuspal deflection that occurs in ETPs. In cases of the absence of coronal dentin walls, post-core application can be applied to increase the retention of the restoration [3]. However, studies have shown that post-space preparation further weakens the remaining tooth structure and increases the risk of tooth fracture or root perforation [8,9]. In cases where the tooth undergoing root canal treatment needs to be kept under observation for a time or the healing of the periapical lesion needs to be evaluated, the intracoronal restorative materials/fiber option can be more appropriate than indirect restoration or intraradicular post-core restorations. The removal of endodontic fiber posts and indirect restorations can be challenging and could weaken the tooth’s structure [10]. The restoration of a tooth with direct resin composites allows it to be done without sacrificing the tooth’s structure or over-preparation. In addition, this technique is relatively more economical and less time-consuming than indirect techniques [11].

Resin composites are frequently preferred as direct restoration materials due to their ability to bond to tooth structures, their aesthetic properties, and their elastic modulus close to dentin [12]. Flowable resin composites are favored for their low viscosity, which allows clinicians to easily restore the teeth; however, one of the major shortcomings of these materials, which have a considerably lower filler than condensable resin composites, is their inherently lower polishability and irregular surface morphology [13]. As a result, a new class of flowable materials named injectable composites have been introduced to the market. These materials exhibit better mechanical properties than those of the initially produced flowable resin composites and provide a better marginal adaptation than condensable resin composites as they retain the ability to spread uniformly [14]. However, choosing the appropriate restoration for endodontically treated teeth with high structural losses is challenging, and regaining the lost strength by applying direct resin composite restorations is debated.

The properties of pre-impregnated fibers for the reinforcement of composites and the enhancement of the flexural strength of fixed or removable polymethyl methacrylate dentures have received increasing attention since the start of their development [15,16]. everStick^®^C&B fibers (GC Corporation, Tokyo, Japan) are pre-impregnated with a light-polymerizable dimethacrylate resin system. These continuous and long fibers contain linear polymer phases that form a semi-interpenetrating polymer network after polymerization. It has been reported that these unidirectional, continuous, long fibers reinforce the teeth as they provide anisotropic mechanical properties [16]. These fibers can also strengthen the teeth when used in different orientations and improve fracture resistance [17]. The orientation techniques used in various studies include transfixing the fiber in the middle of the buccal and palatal walls horizontally, placing the fiber in the bucco-palatal/mesio-distal direction into the cavity, or circumferential application, etc. [18,19].

The fiber reinforcement of dental composites is another improvement that was introduced to enhance restoration toughness and fracture resistance [20]. A short-fiber-reinforced composite (SFRC) has recently been developed for high stress-bearing areas. A SFRC contains millimeter-sized, randomly oriented E-glass fibers and a specific semi-interpenetrating polymer network that provides good adhesion properties to the material [21]. Its short-fiber structure strengthens restorations, prevents crack initiation or propagation, and reinforces the mechanical properties of restorations [22]. everX Flow™ is a flowable SFRC developed as a dentin replacement material. It was launched in 2019 using shorter and thinner fibers to improve its predecessor’s (everX Posterior™) difficult handling features [23].

It is unclear which types of fibers or application techniques should be used to take full advantage of their properties that improve the mechanical performance of a severely damaged tooth. Thus, the effect of different application techniques and fiber types on the direct restoration of endodontically treated teeth should be investigated. Szabo et al. [16] stated that the in case of a clinical situation that allows the use of unidirectional, long, glass fibers, this was the best choice to achieve reinforcement within the structure of restoration in a recent study. To the best of our knowledge, there is no study which compares the effect of different unidirectional, long, glass fiber orientations and short-fiber-reinforced composites on the fracture resistance of ETPs. This study aimed to evaluate the flowable SFRC and long, glass fiber orientations on the fracture resistance of ETPs with MOD cavities restored with an injectable composite. The null hypothesis was that the fracture resistance of direct restorations made using an SFRC or long glass fiber orientation techniques in ETPs with MOD cavities would increase.

## 2. Materials and Methods

The materials, manufacturers, and compositions of the materials used in this study are presented in Table 1.

### 2.1. Tooth Selection

**Figure 1:**
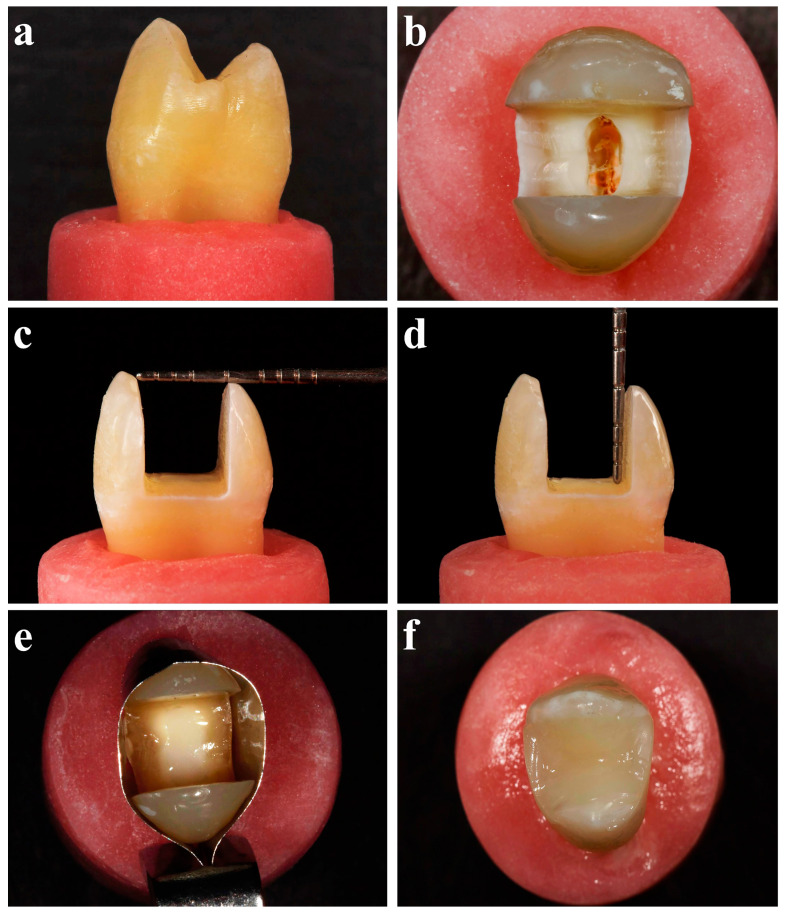
Representative preparation images. (**a**) Teeth were embedded vertically up to 3 mm below the cementoenamel junction into the acrylic resin, (**b**) Gutta-percha was removed 1 mm below the CEJ after the endodontic treatment of the teeth, (**c**,**d**) the measurements of the cavity dimensions were made with a periodontal probe, (**e**) a Tofflemire matrix band was placed around the teeth after the root canal orifices were sealed using a flowable composite material, (**f**) and the cavities were restored with a nanohybrid flowable composite material in a resin composite group.

A total of 90 intact human maxillary double-rooted premolar teeth extracted for orthodontic reasons with similar sizes were obtained from 18–25 year-old patients with approval from the Non-Interventional Clinic Research Ethics Committee of Zonguldak Bülent Ecevit University (protocol number: 2024/1). After removing debris and soft tissue remnants, the teeth were preserved in physiological saline at 4 °C until use. Teeth with restoration or visible crack lines were excluded from the study. The roots were mounted vertically up to 3 mm below the cementoenamel junction (CEJ) into the auto-polymerized acrylic resin (Figure 1a). Endodontic treatment was performed on 75 teeth, while 15 teeth were left untreated as the positive control group.

### 2.2. Access Cavity Preparations and Endodontic Treatment

Standardized endodontic cavities were prepared by a single operator (EH) using an electric motor (NLX Nano electric motor, NSK, Tokyo, Japan) with a round diamond bur (Horico Dental Hopf, Ringleb & Co. Gmbh & Cie, Berlin, Germany) with water cooling. To determine the working length, #10 K files (Dentsply, Maillefer, Ecublens, Switzerland) were inserted into the root canals. The root canals were instrumented with the ProTaper Gold System up to F2 (Dentsply, Tulsa Dental Specialties, Tulsa, OK, USA) and irrigated with 2 mL of 3% sodium hypochlorite between each file. Final irrigations were made with 5 mL of 17% ethylenediaminetetraacetic acid (Sigma–Aldrich, St. Louis, MO, USA) for 1 min followed by distilled water. The root canals were then dried using absorbent paper points, and canal obturation was performed with the single cone technique (PTG F2 gutta-percha point, Dentsply) and a resin paste (AH Plus, Dentsply). Extracoronal excess gutta-percha was removed using a heated plug 1 mm below the CEJ (Figure 1b). The quality of the endodontic treatment was assessed with radiographs. Endodontic cavities were cleaned with alcohol and sealed with a temporary filling material. The teeth were stored at 37 °C and 100% humidity for 7 days.

### 2.3. MOD Cavity Preparations

The temporary filling material was removed and the root canal orifices were sealed using an injectable composite (G-aenial Universal Injectable, GC Corporation, Tokyo, Japan) that was polymerized for 20 s using an LED light curing unit (Elipar S10, 3M ESPE, St Paul, MN, USA). Standardized MOD cavities as described by Soto-Cadena et al. [24] were prepared by the same operator (EH) with an electric motor at 20,000 rpm using cylindrical diamond burs (Horico Dental Hopf, Ringleb & Co. Gmbh & Cie, Berlin, Germany) with water cooling. The cavity walls were prepared parallel to the long axis of the tooth. The measurements of the cavity dimensions were made with a periodontal probe (Figure 1c,d). The measurements were standardized to a bucco-lingual width of 5 mm, a depth of 5 mm, and a mesio-distal width of 6 mm. The thicknesses of the buccal and palatal walls were measured with dental gauge calipers (Iwanson Decimal Caliper, Asa Dental, Bozzano, Italy) and standardized as described by Soto-Cadena et al. [24].

### 2.4. Adhesive Application

The prepared MOD cavities were rinsed with water and dried with an air–water spray. A single operator (AH) applied the same adhesive procedure to the cavities of all specimens. The enamel structure was acid-etched selectively with 37% phosphoric acid for 15 s, rinsed with water, and air-dried. The adhesive (Scotchbond Universal Plus Adhesive, 3M Deutschland GmbH, Neuss, Germany) was applied to the dentin and the etched enamel walls using a disposable applicator and rubbed in for 20 s. Then, the cavity was gently air-dried for 5 s and the adhesive was LED light-cured (Elipar S10, 3M ESPE, USA) for 20 s with light emitted at 1200 mW/cm^2^. After the application of the adhesive, the specimens were divided into five groups according to the different direct restorative techniques to be used.

### 2.5. Resin Composite Group

A Tofflemire matrix band (1101C 0.035, Hawe-Neos, Italy) was placed around the teeth (Figure 1e). The cavities were then restored with an injectable composite (G-aenial Universal Injectable, GC Corporation, Tokyo, Japan) restorative material, which was applied with a 2 mm thickness, consecutively (Figure 1f). Each layer was light-cured from the occlusal surface for 40 s. Aluminum oxide discs (OptiDisc, Kerr, Bioggio, Switzerland) were used for the finishing and polishing procedures for all groups.

### 2.6. Modified Transfixed Technique + Resin Composite Group

**Figure 2:**
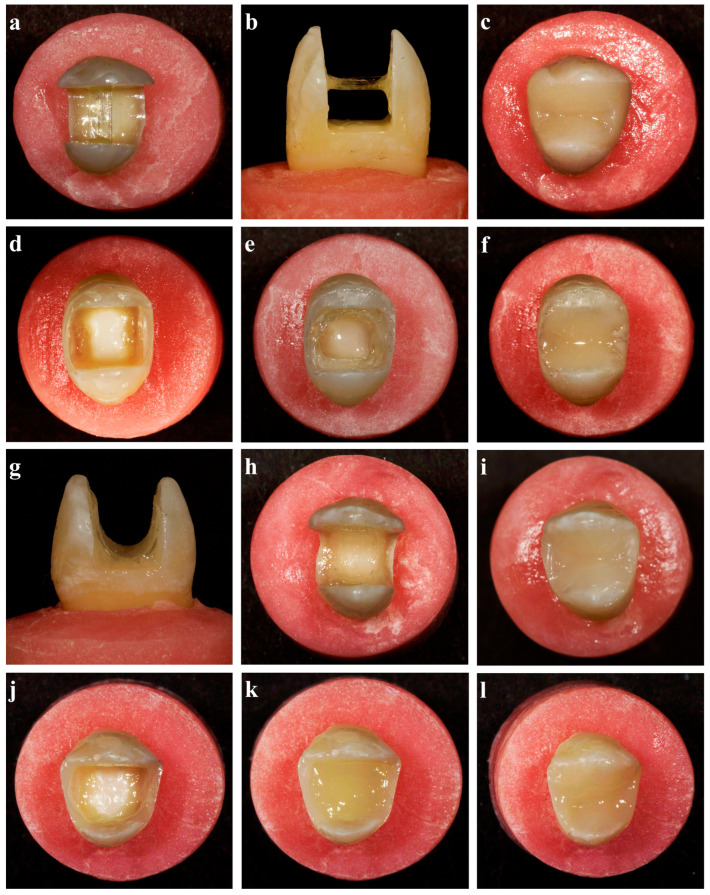
Representative preparation images. (**a**–**c**) The modified transfixed technique + resin composite group; (**d**–**f**) the circumferential technique + resin composite group; (**g**–**i**) the cavity floor technique + resin composite group; (**j**–**l**) the short-fiber-reinforced composite + resin composite group.

A 1.5 mm wide glass fiber strip was cut to the length of the prepared bucco-lingual cavity (5 mm). No additional preparation was made to the cavity walls in which the fiber would be placed or passed through. A very thin layer of an injectable composite (G-aenial Universal Injectable) was applied to the center of the buccal and palatal inner walls (2.5 mm above the cavity floor) where the ends of the fiber strip bond to the cavity wall. The adhesive was applied to the fiber surface, excess adhesive was removed with air–water spray, and the fiber was placed horizontally in the specified area and light-cured (Figure 2a,b). Restorative procedures were performed identically to those of the resin composite group (Figure 2c).

### 2.7. Circumferential Technique + Resin Composite Group

The proximal walls of the tooth were built up with a 1.5 mm thick resin composite by placing a Tofflemire matrix retainer and band around the teeth, before applying the fiber strip (Figure 2d). Preparation for the circumferential technique included the selection of the correct length and width of fiber to properly fit into the cavity. The retraction cord was placed along the inner walls of the cavity as a ring and its length was measured as the guide length. The glass fiber strip was cut to the guide length, which was approximately 10 mm for all specimens. When deviations of more than 0.5 mm occurred, small abrasions were made on the surfaces of the approximal composite walls to keep the length of the fibers constant. A 1.5 mm wide by 10 mm long fiber was prepared and an adhesive bonding agent was applied to the fiber’s surface. After the excess adhesive was removed with air–water spray, the fiber was made into a C shape and placed in the cavity. A very thin layer of injectable composite (G-aenial Universal Injectable) was applied horizontally to the center of the buccal and palatal walls (2.5 mm above the cavity floor) where the fiber strip would be placed. The glass fiber was immediately placed into the uncured injectable composite (G-aenial Universal Injectable) and was then light-cured for 40 s (Figure 2e). Restorative procedures were performed identically to those of the resin composite technique group (Figure 2f).

### 2.8. Cavity Floor Technique + Resin Composite Group

The adhesive was applied to the surface of a 7 mm long and 1.5 mm wide cut glass fiber. An injectable composite (G-aenial Universal Injectable) was applied to the areas where the fiber strip would be placed, which were the middle of the buccal wall, the palatal wall, and the cavity floor. The prepared glass fiber was placed into the cavity in the bucco-palatal direction with its midpoint bonding to the cavity floor and in a way that it would not reach the margins of the cavity (Figure 2g,h). After light-curing the fiber, the resin composite, and the adhesive for 40 s, the cavity was restored as previously described (Figure 2i).

### 2.9. everX Flow™ (SFRC) + Resin Composite Group

The proximal walls were built up with the injectable resin composite (G-aenial Universal Injectable) with a thickness of 1.5 mm by placing a Tofflemire matrix retainer and band around the teeth (Figure 2j). The cavity converted to Class I was filled with an SFRC (everX Flow™, GC, Tokyo, Japan) up to 4 mm above the cavity floor (Figure 2k). The remaining part of the cavity was restored with the injectable composite (G-aenial Universal Injectable) using the horizontal layering technique (Figure 2l).

### 2.10. Fracture Strength Test

**Figure 3:**
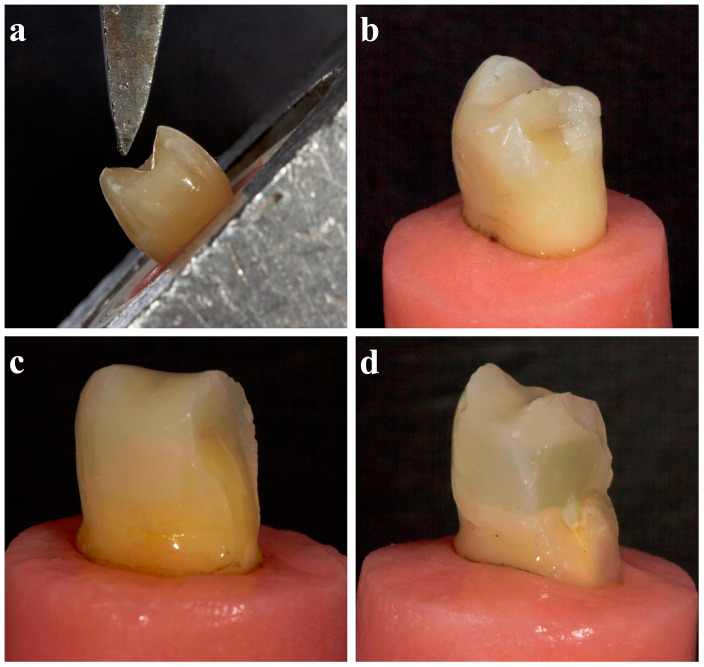
(**a**) Fracture strength test; (**b**) a representative repairable fracture mode image (fractures not extending below the CEJ); (**c**) a representative image of the possibly repairable fracture mode (fractures extending below the CEJ but not below the acrylic line); (**d**) a representative unrepairable fracture mode image (fractures extending below the acrylic line).

All prepared specimens were stored in distilled water at 37 °C until they were used in the fracture strength test. A 30° oblique compressive load was applied to create a single contact in the buccal cusps at the junction of the restoration and enamel using a universal testing machine (PWS-E100, Shimadzu Co., Kyoto, Japan) at a crosshead speed of 0.5 mm/min until fracture occurred (Figure 3a). The load in the control group was applied at a similar location to that of the other groups. The type of fracture pattern was determined via visual inspection under ×40 with a stereomicroscope (EZ4W, Leica Microsystems, Milton Keynes, UK) and was classified as repairable (fractures not extending below the CEJ; Figure 3b), possibly repairable (fractures extending below the CEJ, but not below the acrylic line; Figure 3c), and unrepairable (fractures extending below the acrylic line; Figure 3d) [23]. The frequency percentages of the fracture patterns were recorded.

### 2.11. Statistical Analyses

Data were analyzed with IBM SPSS V23 software (Chicago, IL, USA). The conformity of the values to a normal distribution according to the groups was examined using the Shapiro–Wilk test. The comparison of fracture strength values according to the groups was analyzed using a one-way analysis of variance (ANOVA). Tukey’s honest significant difference post hoc test was used to analyze the differences between the groups. Pearson’s chi-squared test was used to compare the repairability of the groups after fracture. The significance level was set at 0.05.

## 3. Results

The mean and standard deviation fracture strength values of the groups are presented in Table 2. Fracture strength values differ according to the groups (*p* < 0.001). The highest mean fracture strength values were obtained in intact teeth/the control group (599.336 N), followed by the modified transfixed technique + resin composite (496.58 N), SFRC (EverX Flow™) + resin composite (469.62 N), resin composite (443.51 N), circumferential technique + resin composite (442.835 N), and cavity floor technique + resin composite (404.623 N) groups, respectively (*p* < 0.05). There was no significant difference between the mean fracture strength values of the resin composite and the circumferential technique + resin composite groups (*p* > 0.05).

**Figure 4:**
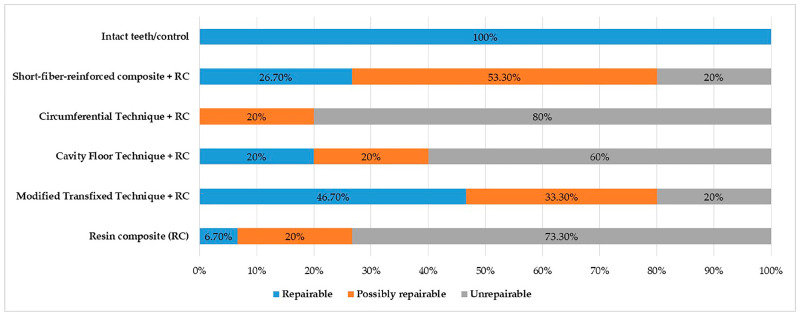
The fracture mode distribution for each group (*p* < 0.001).

The fracture pattern distribution for each group is presented in Figure 4. Repairability was found to be dependent on the restoration method (*p* < 0.001). Among the groups, the highest percentage of specimens that were repairable after fracture was that of the intact teeth/control (100%) and the modified transfixed technique + resin composite (46.7%) groups, while the lowest percentage was observed in the circumferential technique + resin composite group (0%). The highest percentage of unrepairable specimens was observed in the circumferential technique + RC (80%), resin composite (73.3%), and cavity floor technique + resin composite (60%) groups, respectively. The highest percentage of possible repairable specimens was observed in the SFRC group (53.3%).

## 4. Discussion

For the successful restoration of teeth, the restoration material is required to bond to the tooth structure, regain the lost fracture resistance, and strengthen the tooth by acting as an internal splint [16]. Double-rooted maxillary premolars were selected in this study because they become more susceptible to fracture under occlusal loading than posterior teeth due to their complex root anatomy and the inclination of the cusps [11]. When restoring such weakened teeth, more materials or techniques should be investigated to prevent and stop the spread of the cracks and fractures that can occur as a result of occlusal forces on the remaining tooth structure. In this study, it was observed that the fracture strength decreased statistically significantly in all restored groups compared to that of the intact teeth.

Over the past 20 years, the use of polyethylene fibers (Ribbond THM; Ribbond Inc., Seattle, WA, USA) for direct restorative techniques with various orientations has been evaluated in numerous studies [17,19,25,26]. Some studies report that, regardless of their orientation, polyethylene fibers form a stress-absorbing layer, preventing possible cracks and fractures and increasing fracture strength [17,25,26]. On the other hand, glass fibers contain interlaced glass, which is an amorphous material that consists of bonded tetrahydrate silica in a random lattice; therefore, glass fibers have varying physicochemical properties differentiating them from polyethylene fibers [27]. Glass fibers are frequently used materials for restorations, similar to polyethylene fibers because they increase the impact strength of composites [28]. Also, some studies state that the reinforcing effect of glass fibers is greater than that of polyethylene fibers [28,29]. However, there are limited studies on the effect of glass fiber orientations on fracture resistance. Therefore, glass fiber orientations were investigated in this study.

In this study, it was found that the effect of everStick^®^C&B on fracture resistance was variable when applied in different orientations. This result is consistent with the study by Sary et al. [17], which investigated the fracture strength of both polyethylene fiber and glass fiber orientations. According to our results, direct resin restorations using the modified transfixed technique and everX Flow™ (SFRC) increased the fracture strength of the ETPs. However, the circumferential technique had no significant effect on the fracture strength, while the unidirectional glass fiber placed on the cavity floor decreased the fracture strength of the ETPs. Therefore, the null hypothesis was partially accepted.

The transfixed technique is described as creating grooves in the buccal and palatal/lingual cusps to horizontally insert the post [30], which has also been labeled as the transcoronal [17] or the horizontal fiber post [31] technique. In the studies where this technique was used with different materials, such as polyethylene fibers [17], a glass fiber post [32], or a zirconia post [30], it was found that this technique increased the fracture resistance of teeth. To our knowledge, there are no studies that have used everStick^®^C&B with this technique. Szabo VT et al. [16] reported that this material increased the fracture strength of direct composite bridges when inserted into the restoration. In this study, the measured fracture strength in the modified transfixed technique using everStick^®^C&B was found to be higher than in other techniques. The long, continuous, and unidirectional E-glass fibers provide great reinforcement against forces perpendicular to their long axis, thus, a possible reason for this result might be that everStick^®^C&B is strong when stretched due to the inherent properties of the material [16]. Burke et al. [33] reported that in a MOD preparation, the pulp chamber roof is the closest link between the cusps and distributes the masticatory and functional stresses. As such, it is possible that the fiber strip bonded between the cusps distributes the occlusal forces similarly to the pulp chamber roof, thus increasing the tooth’s resistance to stresses and reducing cuspal deflection. In this study, unlike other post systems used for the transfixed technique mentioned above, everStick^®^C&B was cut to the required width between the buccal and palatal inner cavity walls, owing to its flexible structure, and bonded without groove preparation. With this technique, the further loss of the structural material required for groove preparation was avoided. We refer to the more conservative technique used in this study, “modified transfixed”, and assume that it has a positive effect on fracture strength. With this technique, applying a packable composite after the fiber is bonded could be difficult in terms of manipulation. In this study, this limitation was overcome by using an injectable composite. Nevertheless, despite the restorative procedures, core build-up materials show different performances and the choice of the material should be a well-considered decision [34]. Since the materials and preparation techniques used are different, no study directly compares to the results of this study. However, these findings are consistent with a recent systematic review and meta-analysis by Abdulrab et al. [31], which investigated the effect of horizontal glass fiber posts on fracture strength.

Several studies have reported varying results on the effect of direct composite restorations on fracture strength when examining fiber placement techniques at the cavity floor. In most of these studies, a polyethylene fiber was used and there are few studies where glass fibers were applied. In some of these studies [26,35,36], it was stated that a polyethylene fiber placed bucco-lingually on the cavity floor increased the fracture resistance of the restoration, while others [25,37] reported no significant difference. Although the results cannot be compared directly because different materials were used, contrary to the aforementioned studies, bonding glass fiber bucco-lingually on the cavity floor before resin composite application reduced fracture resistance. In this study, due to the structure of the used glass fiber, it was not laid in the cavity, it was placed in contact with the walls with the injectable composite at the buccal, palatal, and cavity floor during the preparation of cavity floor techniques. Although it is possible that the fiber is more stretched against the occlusal force compared with the aforementioned technique (laying inner walls of the cavity), the low fracture strength of this technique might be due to the shape memory of the material. In one of the few studies to investigate the orientation of glass fibers, Sary et al. [17] compared the effect of the placement of a bidirectional glass fiber net (everStick^®^NET, GC) in different directions and found that its placement at the floor of the cavity in the bucco-lingual direction had worse fracture strength values than its circumferential or occlusal placement, similar to our results.

Another fiber reinforcement technique is the circumferential application of fiber to the inner cavity wall surfaces [17,25,38], also known as wallpapering [19,36] or circular orientation [27]. Studies report that polyethylene fibers [17,26,36] or glass fibers [17,38] increase the fracture resistance of direct composite restorations when applied using the circumferential technique. Contrary to the results of these studies, in our study, it was found that the long glass fiber did not have a significant effect on fracture strength when applied with the circumferential technique compared with the resin composite group. Similar to our result, Akman et al. [25] reported that this technique had no significant effect on fracture resistance. Sary et al. [17] also reported that higher fracture strength values were obtained with the circumferential technique than with composite restorations. When comparing these results, it should be taken into account that everStick^®^NET and everX Posterior™ (SFRC) were applied together in the mentioned study. Regarding this, the authors reported that even when SFRC was used alone as a dentin substitute, an isotropic strengthening effect of randomly oriented fibers was possible in multiple directions rather than a few specific directions [39]. In this study, unidirectional glass fiber was restored with an injectable composite without an SFRC, and it was found that a circumferential fiber application did not make any additional contribution to the fracture strength of the composite. In another study, Navimipour et al. [38] investigated the fracture resistance of ETPs when applied occlusally, circumferentially, or both, using a different glass fiber material (Interlig; Angelus, Londrina PR, Brazil). They reported that fiber use increased the fracture strength of resin composite restorations. In the current study, no statistically significant increase was found when using the circumferential technique. This result might be due to the different materials used or the varied direction of the force applied during the test (parallel to the long axis of the teeth).

Studies have reported that when everX Flow™ is used as a dentin replacement material, it provides a higher fracture resistance compared to that of teeth restored with a resin composite that is not reinforced by a short fiber [32,36,40,41]. The results of this study also support this finding. A recent clinical study also stated that the application of an SFRC, without proximal surface coverage in Class II restorations, yielded satisfactory clinical outcomes throughout the 18-month follow-up [42]. everX Flow™ has been reported to have a higher fracture toughness (2.6 MPa m^1/2^) than resin composites (1.1–1.9 MPa m^1/2^), resulting in higher structural performance and fatigue strength [40]. Since the fibers are oriented randomly in SFRCs, the mechanical properties and the ability for potential reinforcement are equal and three-dimensionally isotropic in all directions. In our study, the group that used everX Flow™ as a dentin replacement material showed higher fracture strengths than all groups except for the modified transfixed technique. Similarly, a recent study reported that polyethylene fiber showed a lower fracture strength than everX Posterior™ when applied to the floor of the cavity [24]. In contrast, Abdulamir et al. [36] reported that polyethylene fiber orientations (via the circumferential technique or being placed on the cavity floor) showed higher fracture strength values than everX Flow™. Agrawal et al. [35] also reported that a polyethylene fiber provided better fracture strength values than everX Posterior™ when placed at both the gingival and pulpal floors and that there was no difference when it was placed in the bucco-lingual or mesio-distal direction. These differences could be due to the application of different types of fibers or the direction of the force applied during the fracture test.

Considering various types of fractures, more unrepairable fractures were observed in all restored teeth when compared with intact teeth. According to the results of this study, the types of fractures in long-glass fiber applications differed depending on their orientation, as in the fracture strength. Unrepairable fractures were observed mostly in the groups where fiber was applied to the circumferential and cavity floor. Most repairable fractures were observed in the modified transfixed group among the experimental groups. The SFRC contributed to the durability of restoration by reducing the irreparable fracture mode compared with that of the injectable resin composite group.

The limitations of this study are that the biomechanical properties of the periodontium were not simulated in the oral environment and the static loading conditions did not replicate the clinical situation. Another limitation of this study was the shape memory of the everStick^®^C&B, which made it difficult to apply the fiber with the circumferential and cavity floor technique. When considering the use of these techniques in clinical situations, bending this material and applying it to narrow cavities may be challenging, especially in the posterior regions. Future studies are needed to compare the modified transfixed technique applied with long glass fibers, which can be prepared more conservatively with the horizontal post technique. Moreover, the evaluation of stress distribution at the cuspal deflection through different methods, such as finite element analysis, that help to evaluate the effect of combined factors on the tooth-restoration complex and to understand strain distributions is suggested for future research.

## 5. Conclusions

Within the limitations of this in vitro study, the following conclusions have been drawn:

1. Direct restorations using unidirectional long fibers with modified transfixed technique or flowable SFRCs increase the fracture strength of ETPs.
2. In the resin composite restorations of endodontic, treated posterior teeth, bonding everStick^®^C&B between the buccal and palatal cusps with the modified transfixed technique could be a conservative treatment option to improve the decreasing fracture strength.
3. From a clinical perspective, using flowable SFRCs was easier and less time-consuming compared with applying long fiber orientations.
4. everStick^®^C&B bonded between cusps horizontally or everX Flow™ applied as a dentin replacement material improves the fracture pattern of direct resin composite restorations.

## Figures and Tables

**Table 1:** Materials used in the study.

Material	Manufacturer	Fillers	Matrix
G-aenial Universal Injectable, flowable composite	GC Corporation, Tokyo, Japan (Lot number: 2103192)	Silicon dioxide (SiO_2_), barium glass, 69 wt%, 50 vol%	UDMA, Bis-MEPP, TEGDMA,
everX Flow™, short-fiber-reinforced composite	GC Corporation, Tokyo, Japan (Lot number: 2106221)	Micrometer scale glass fiber filler, barium glass, 70 wt%, 46 vol%	Bis-EMA, TEGDMA, UDMA,
Scotchbond Universal Plus Adhesive	3M Deutschland GmbH, Neuss, Germany (Lot number: 7730432)	Bis-GMA, 10-MDP, 2-HEMA, Vitrebond copolymer, ethanol, water, initiators, fillers methacrylate, water	
everStick^®^C&B fibers	GC Corporation, Tokyo, Japan (Lot number: 2210201)	Silanated unidirectional glass fibers	PMMA, Bis-GMA

**Table 2:** The mean and standard deviation fracture strength values of the groups. a–e: Different lowercase letters indicate significant differences between groups.

Groups	Mean ± sd (N)	Test Statistic	*p*
Resin composite (RC)	443.511 ± 19.17 a	135.383	<0.001
Modified transfixed technique + RC	496.58 ± 19.67 b		
Cavity floor technique + RC	404.623 ± 19.21 c		
Circumferential technique + RC	442.835 ± 31.89 a		
Short-fiber-reinforced composite + RC	469.62 ± 19.56 d		
Intact teeth/control	599.336 ± 22.9 e		
Total	476.084 ± 65.9		

## Data Availability

The data presented in this study is available in this article.
